# Finding the Way with a Noisy Brain

**DOI:** 10.1371/journal.pcbi.1000992

**Published:** 2010-11-11

**Authors:** Allen Cheung, Robert Vickerstaff

**Affiliations:** 1 Queensland Brain Institute and School of Information Technology and Electrical Engineering, The University of Queensland, Brisbane, Australia; 2 AgResearch Ltd, Christchurch, New Zealand; University College London, United Kingdom

## Abstract

Successful navigation is fundamental to the survival of nearly every animal on earth, and achieved by nervous systems of vastly different sizes and characteristics. Yet surprisingly little is known of the detailed neural circuitry from any species which can accurately represent space for navigation. Path integration is one of the oldest and most ubiquitous navigation strategies in the animal kingdom. Despite a plethora of computational models, from equational to neural network form, there is currently no consensus, even in principle, of how this important phenomenon occurs neurally. Recently, all path integration models were examined according to a novel, unifying classification system. Here we combine this theoretical framework with recent insights from directed walk theory, and develop an intuitive yet mathematically rigorous proof that only one class of neural representation of space can tolerate noise during path integration. This result suggests many existing models of path integration are not biologically plausible due to their intolerance to noise. This surprising result imposes significant computational limitations on the neurobiological spatial representation of all successfully navigating animals, irrespective of species. Indeed, noise-tolerance may be an important functional constraint on the evolution of neuroarchitectural plans in the animal kingdom.

## Author Summary

The ability to navigate allows animals to vastly increase the action space for finding resources, mates, and to avoid predators. The benefits are many and it is commonly believed that modern brain functions have emerged from ancestral forms evolved for effective navigation. Since the time of Charles Darwin, it has been recognized that path integration is a navigation strategy innate to many species. Path integration involves adding the stepwise displacements during a circuitous journey to compute a net homeward direction. Over the past century, this phenomenon has been described for birds to mammals to arthropods, and a long list of mathematical, algorithmic, and neural network models have been proposed to explain the necessary computations. This work shows how the different types of models behave in the presence of noise. It turns out that only one class of models can function properly in the presence of noise. Since noise appears to be present at all levels of brain physiology, we arrive at the surprising conclusion that the general computational principles for path integration must be the same across all species. Two subtypes of path integration models share the same critical computational principles, and are compared to known neuroanatomy and physiology.

## Introduction

In nature, successful navigation is vital for survival. It follows that neural circuitry capable of carrying out navigation must be ubiquitous in the animal kingdom. The study of animal navigation, therefore, is not only important in its own right, but may offer general insights into the architecture, computational algorithms and evolutionary history of modern nervous systems.

### Path Integration

For convenience and consistency with previous work, we use the term ‘navigation’ in a general sense to encompass all forms of non-random locomotion, including biological *path integration* (PI) or ‘dead reckoning’, a process described by Charles Darwin in 1873 [1]. Darwin realized that documented feats of navigation amongst the local inhabitants of Northern Siberia were likely to have been achieved by mentally keeping track of the changes in heading and distances travelled. This observation was significant as it distilled navigation into a concise computational problem which could be tested experimentally and formalized mathematically. PI is arguably the simplest navigation strategy which requires a neural representation of space. In contrast, strategies such as chemotaxis or view-based homing, although biologically significant, do not necessarily allow us to probe at the neural representation of space.

Since Darwin's time, much knowledge has accrued about the neuroethology of navigation, including PI and landmark-based navigation. With advanced *in vivo* recording and measurement techniques, a number of likely neuronal correlates of navigation have been identified [2]–[6]. Despite a plethora of data, it is still unclear even in principle how animals represent space, especially across the phylogenetic expanse. In fact, it is completely unknown whether there is any underlying reason for different species to obey the same rules. PI seems to be an ideal process for investigating the neural representation of space since it maintains a continual record of position in space. Systematic probing of this record could theoretically define the complete mapping between real and representational space. Furthermore, PI-related behaviour has already been documented in a wide variety of animal species [see 7], and it seems plausible that some sort of PI system may exist in most nervous systems capable of navigation. Finally, a consistent representation of space may simplify the computations necessary for combining different navigation strategies to generate a single coherent output. This supports the hypothesis that the entire neural representation of space is likely to be the same as that used for PI, based on the principle of reusing existing circuitry as well as computational parsimony. Such arguments have specific biological and modelling implications in light of the theoretical results of this work, and will be discussed further below.

### A Neural Representation of Space

Tolman's cognitive map may be the first serious theoretical formulation of the spatial foundation for navigation in any animal [8]. More than two decades elapsed before the discovery of hippocampal place cells [2], which have widely been considered to be the neurophysiological correlates of the ‘cognitive map’. The more recently discovered medial entorhinal grid cells [5], [6] have already gained a remarkable level of agreement to be the neural substrate of mammalian PI [6], [9]–[13]. In contrast, neural correlates of arthropod navigation have been difficult to find, in part due to technical limitations. Nonetheless, lesion experiments suggest the mushroom bodies of cockroaches may serve similar navigational functions as the mammalian hippocampus [14]. Furthermore, the central complex of the locust has a topographical architecture with directional tuning [15], functionally reminiscent of the rodent head direction system [16].

In the arthropod literature, a vast body of behavioural evidence exists for the use of PI as a fundamental strategy of navigation, but concurrent neurophysiological data are lacking. In mammals, there is an abundance of place cell and grid cell data, showing firing fields which strongly correlate with spatial locations. These are, *prima facie*, the best neuronal correlates of spatial representation known. However, most of these data are obtained from animals navigating in artificial, relatively simple and spatially restricted arenas. Furthermore, the relevance of these cells has been called into question in a variety of navigation paradigms [17]–[19]. It is unclear whether strong conclusions can be drawn from either arthropod or mammalian data with respect to the true nature of the neural representation of space. Moreover, experimental and behavioural data on *what* exists in nature does not necessarily answer *why*. Our work addresses this important question by gaining an in-depth theoretical understanding of whether PI places any constraints on a neural representation of space. It turns out that the single assumption that all nervous systems (including biological PI systems) are susceptible to noise, is sufficient to differentiate existing PI models on a functional/behavioural basis.

### Neural Noise: A Problem and Solution

It has been shown that under ideal, noise-free conditions, a range of mathematical and neural models of PI are quantitatively equivalent for updating trajectories through metric space, and that the equivalence could be extended to descriptions of steering, searching behaviour and even account for observed systematic errors [7]. This is unambiguous theoretical validation of the wide range of models which have emerged as candidates for arthropod PI. However, since the alternative models behave equivalently, they have equal explanatory value. Is there any principled way of differentiating between the models?

To properly answer questions about animal navigation, we need to build an understanding of species-independent truths about neurobiological spatial representations. Here, we approach this problem from a theoretical perspective. From first principles, we show how different PI systems will behave in the presence of noise. Using a general classification scheme for spatial representations during PI [*7*], we show how imperfections or noise in different neural representation of locomotion results in distinct outcomes, corresponding to two distinct types of *directed walks* [20], [21]. Only one type of directed walk, and hence the corresponding neural representation of space, can faithfully capture the real trajectory using PI. The other representations yield irrecoverably large errors, rendering the PI system useless beyond a few steps. Finally, we apply our understanding of directed walks to discuss the implications of the results on the neurobiology of PI and navigation.

## Materials and Methods

The results presented in this work can be understood as a mapping of the results of directed walk (DW) theory from a walk carried out in physical space to a walk or sequence of representational states taking place within the nervous system of an animal navigating by PI. The results section introduces the details of the mapping process, and establishes a strict equivalence between the physical and representational walks. The conclusions then follow automatically from the previously demonstrated results of DW theory. Of the two types of DW, only one can tolerate noise without quickly degenerating to the point where the animal is lost. Of the four classes of spatial representation, only one is equivalent to the robust type of DW and is therefore the only class tolerant of noise during PI.

The theoretical insights arise from the combination of 1) the generally accepted assumptions of sensorimotor noise and process noise within the nervous system, 2) theory of directed walks, and 3) a recently developed classification scheme for PI systems. The contributions of each component to the final results are explained and justified next. Some technical details have been omitted for clarity but can be found in the supplementary material and listed references.

### Noise in Biological Neural Networks

Noise from the environment, the internals of a navigating agent, and the interface between the two, can all contribute to positional uncertainty during a navigation task. It has been demonstrated using simulations [22] and theoretical proofs [20], [21] that the type of directional cue used for PI is critical i.e., an external compass is a necessity for successful PI. Here we focus on the PI system *per se* rather than purely sensory or motor noise (which of course are inevitable). The motivations and mathematical implementations are described below.

Biological noise can arise from a variety of sources within a nervous system [23]. From neurotransmitter diffusion, to ion channel kinetics, to action potential timing, stochastic behaviour appears to be pervasive throughout biological neural networks. Nevertheless, there is also growing evidence that neural systems have evolved near optimal systems-level solutions to common problems, even where optimal solutions may seem implausibly complex in explicit mathematical terms [24]. Therefore we ask whether certain types of neural representations of space may be superior in some way, in the presence of noise. In this work, we consider two major sources of noise, namely sensor noise (*δ*) which leads to imperfect inputs, and representational noise (*ε*) which manifests itself during the updating step of PI. We do not assume specific characteristics about the noise, but simply that it exists. In essence, we algebraically corrupt the input and updating processes with noise and consider the effects. While noise tolerance is obviously a key issue for any biological navigation system, the relevance of our results to non-biological systems is not so clear. Robotic systems commonly use representations allowing a very high degree of precision (typically 16 significant places or more) which are generally operated on by algorithms which do not introduce any random errors during processing. These are the kinds of conditions under which the fundamental mathematical equivalence between all spatial representations might lead to identical performance regardless of the spatial representation used [7]. Nonetheless, sensorimotor noise or rounding errors may still differentially affect the performance of non-biological spatial representations, especially for long journeys or where extreme precision is required, but their properties are subtly different to their biological counterparts (rounding errors are not truly random, sensory values can be sampled once and stored for future access without subsequent degradation) which are beyond the scope of the present account. Here we focus on navigation in the context of biological nervous systems where machine-level precision is implausible and where, even in the absence of noisy sensory data, the addition of noise by the representational system leads to positional uncertainty.

Mathematically, we model two types of independent, random errors which are assumed to accrue during PI. It is of course true that noise may arise anywhere in a neural network subserving PI. Indeed, every computational and/or network variant will accrue noise in subtly different ways, leading to quantitative variations in the magnitude of PI errors. For instance, an update algorithm which requires multiple feedback steps, particularly if signal integration is involved, may well result in greater susceptibility to noise. This idea was the basis of an argument that polar representations are computationally inferior to Cartesian ones [25]. However, this type of reasoning contains at least three weaknesses: firstly, an explicit PI update algorithm needs to be assumed for each spatial representation in order to quantify the number of feedback loops, and therefore susceptibility to noise. For instance, what if the neural implementation of a Cartesian PI system actually required many more computational steps than the explicit mathematical version? Secondly, even if all mathematical operations had simple counterparts in neurobiology, it is still unclear whether a polar representation could consistently outperform a Cartesian one simply by having smaller error magnitudes. Thirdly, do these computational arguments apply to variants of PI models which are neither strictly Cartesian nor polar?

We avoid these problems in two ways: firstly, we use an extended classification system for spatial representations; secondly, we do not explicitly assume any particular computational algorithm except what is directly implied by the classification system. Instead, we assume that there is a minimum amount of noise which corrupts the PI update process, at each step of the journey. The details of the noise are described below.

Every allocentric heading, 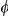, and rotation measurement Δ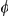 is associated with an error term *δ*. Even if the ideal Δ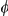 is zero (no rotation) a finitely small amount of noise *δ* corrupts the signal. Since it is impossible for the PI system to “know” that there was no rotation, the best it can do is assume the input 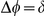. Generally, perfect compass or rotational inputs are impossible. Furthermore, the state of the path integrator is assumed to be updated following every step and that each state parameter which is updated is associated with an error *ε*. Thus the updating process is assumed to be imperfect.

Quantitatively rigorous results have been reported to describe the way in which cumulative noise affects navigation using idiothetic or allothetic directional cues [20], [21], as outlined in the next section. The resulting behaviours are vastly different depending on the directional cue, suggesting a mathematical framework for distinguishing between different classes of movement trajectories. Neural representations of real trajectories may be considered in the same way. Geometric constructions will be developed in this work to map trajectories in real space to representational space, via the process of PI. The logic of this mapping is critical for understanding the theoretical findings of the current work.

### Directed Walk Theory

**Figure 1:**
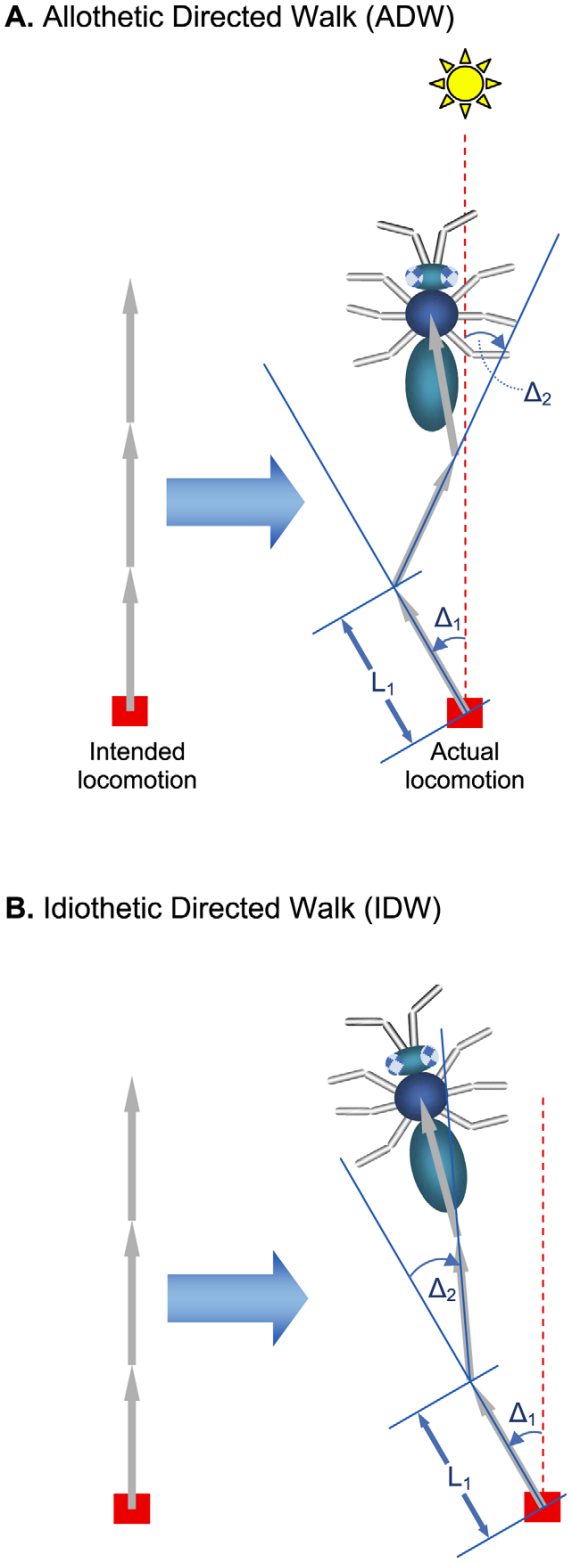
Illustration of simple directed walks. (**A**) An allothetic directed walk (ADW) occurs when the navigating agent measures current heading directly from an external reference (a compass, represented here as a sun). Following each step, the agent is able to reorient itself to the desired compass direction. (**B**) An idiothetic directed walk (IDW) occurs when the navigating agent estimates current heading by integrating rotations, often estimated from internal cues. Without a compass, the agent cannot reorient itself following each step. The illustrative animal is an arthropod consisting of a head, thorax (assumed to be the point-position of the animal for illustrative purposes), and abdomen. In both cases, the animal intends to take three steps away from home (red rectangle) ideally along a straight line (intended locomotion), but due to cumulative sensorimotor noise, moves along the actual trajectories as shown. For illustrative convenience, the sun is aligned with the direction of intended locomotion, which is designated the X-axis in the text. See text for further details.

At its simplest, a directed walk (DW) consists of a sequence of discrete movements or steps, 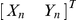, all intended to be in the same direction (which for convenience and consistency with previous notation, is designated as the *X*-axis, and is oriented vertically in Fig. 1). Ideally, without noise, at step *n*, the animal moves by 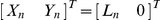. However, due to unavoidable noise, the animal makes a rotational error and a distance error (Eq 1). DW theory shows how errors accumulate during such a journey [20], [21].

In principle, the basic unit of locomotion of DWs should reflect the anatomy and physiology of the locomoting animal. The mathematical description can therefore range from a simple elementary step [20], to a general biased elementary step [21]. In the main text, we use simple elementary steps for clarity, whose formal description consists of just an unbiased turn error (denoted as a standalone 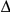, distinct from the prefix meaning change) and a step length *L* whose linear error is independent of 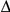
_._ Note the physical turn error 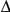 in real space is the unavoidable final output error and is conceptually distinct from a measured rotational error *δ* which represents an unavoidable input error (explained earlier).

Note that the conclusions still apply even when these simplifying assumptions are relaxed to a general biased elementary step (which accounts for any locomotory pattern, error distribution, and statistical dependence between step components - see supplement, and ref [21]). This is necessary to generalize the proof to allow for any realization of each class of spatial representation. It should be noted that DWs account for errors in the step length as well, but previous results showed these are only of secondary importance compared to the angular error 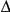 except for very short journeys [20], [21]. Although not discussed in the following analysis, it is understood that *L* incorporates some random error.

Of fundamental importance to an understanding of the effect of cumulative noise in real space is the distinction between two types of directional information during navigation. This distinction turns out to be the critical determinant of the type of DW which results when an animal attempts to move in a straight line [20], [21].

One type of directional cue, like a geomagnetic compass, is an absolute external (allothetic) directional reference which is available continuously. Using a compass or other allothetic directional cue to move from point A to point B results in an *allothetic directed walk* (ADW, Fig. 1A - subtleties about what constitutes a compass, how a compass should be used, or fine distinction between allothetic and idiothetic cues are discussed elsewhere [21]). During an ADW, the displacement at step *n*, expressed in a Cartesian reference frame, can be written as
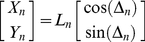
(1)The alternative type of directional cue, where direction is estimated by internally accumulating measured rotations, is termed idiothetic. Using idiothetic directional information in moving from point A to point B results in an *idiothetic directed walk* (IDW, Fig 1B). The displacement of step *n* is thus
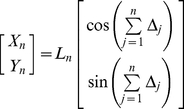
(2)Note that the total angular displacement error for each step *n* is the sum of all angular errors up to and including that of step *n* (in contrast to an ADW, Eq 1).

It is now well understood that the properties of ADWs and IDWs are qualitatively and quantitatively different in the presence of any noise, or error. Two important differences between ADWs and IDWs are briefly outlined. Firstly, the expected (mean) position of an IDW has a finite limit, irrespective of how many steps are taken [20], [21]. Recently, this limit was shown in walking humans to be approximately 100 m [26]. In contrast, there is no such limitation for an ADW. Secondly, the positional uncertainty (variance) of an IDW is generally greater than that of a pure random walk, which in turn is greater than following an ADW [20], [21]. In other words, an IDW leads to nonlinear systematic errors coupled with large random errors in position, whereas an ADW results in linear systematic errors coupled with small random errors in position. Assuming a biologically plausible level of noise, the positional uncertainty following an IDW becomes so large that the navigating agent is lost, even after very few steps. Under the same noisy conditions, following an ADW, the navigating agent is positioned close to the ideal location with relatively very little uncertainty. Importantly, the average trajectory of an ADW has the same shape as the ideal trajectory, only smaller by a constant factor.

If we assume that accurate PI is biologically advantageous, then we would predict that natural selective pressures would favour the development of a spatial representation with minimal error. For instance, given a simple straight trajectory in physical space, a trajectory which resembled an ADW in representational space would have smaller errors and be a more faithful representation than one which resembled an IDW.

During locomotion, from one step to the next, what distinguishes an ADW from an IDW? It turns out that the essence of this problem can be distilled down to one fundamental question – do angular displacement errors accumulate? If angular displacement errors accumulate, then the behaviour resembles an IDW (illustrated in Fig. 1B), otherwise an ADW (illustrated in Fig. 1A; summarized in Table S1 for general locomotion).

### Classifying Neural Representations of Space

**Figure 2:**
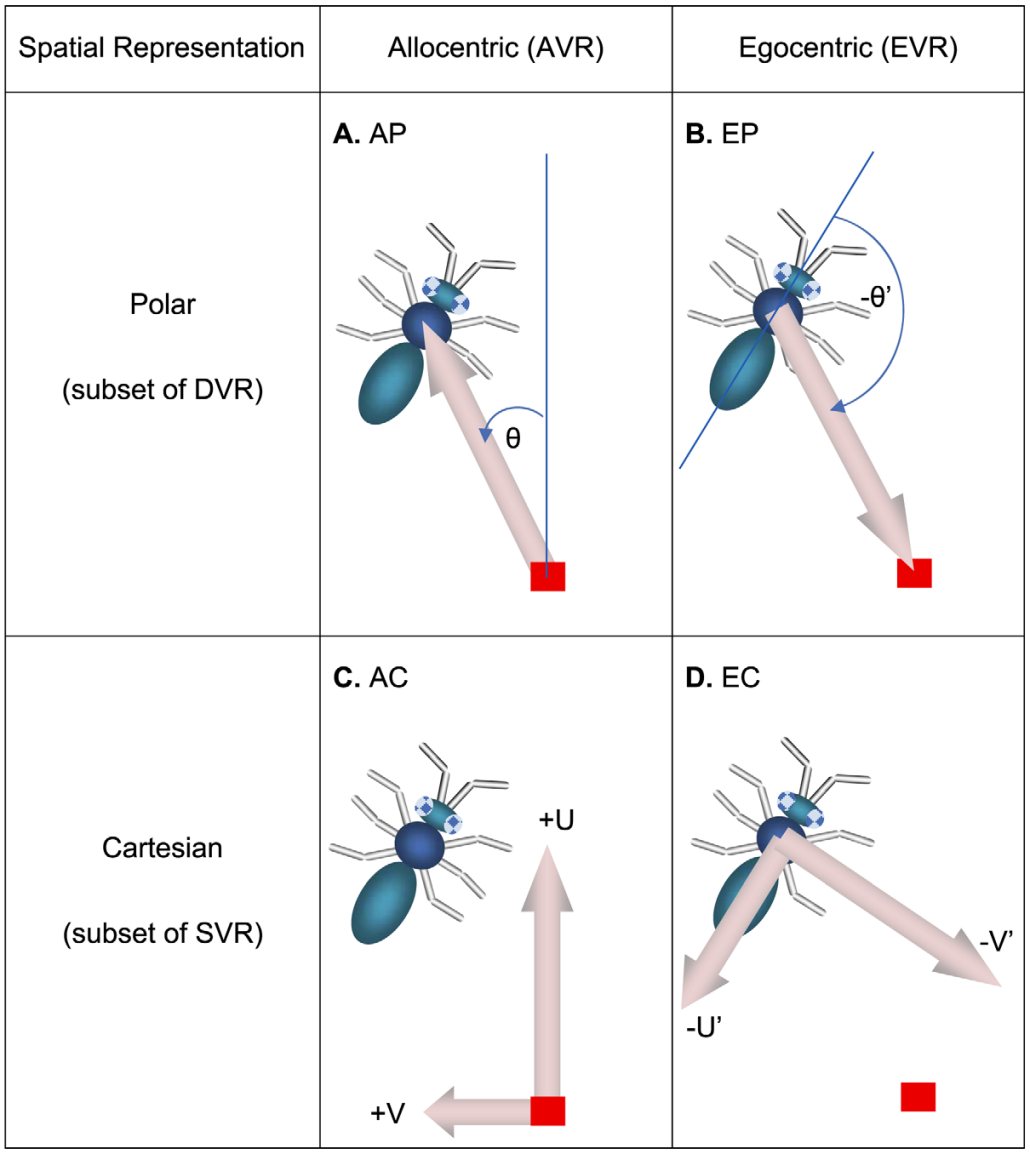
Examples from 4 extended classes of neural representations of 2-D Euclidean space. (**A**) An allocentric polar (AP) representation as an example of an allocentric dynamic vectorial representation (ADVR). (**B**) An egocentric polar (EP) representation as an example of an egocentric dynamic vectorial representation (EDVR). (**C**) An allocentric Cartesian (AC) representation as an example of an allocentric static vectorial representation (ASVR). (**D**) An egocentric Cartesian (EC) representation as an example of an egocentric static vectorial representation (ESVR). In representational space, the direction of intended motion is denoted +U (analogous to +X of real space - see Fig 1). Similarly, +V is analogous to +Y. By convention, it is assumed that egocentric “forward” is rostral (+U′), “backward” is caudal, leftward is +V′ and rightward is −V′. The thick pink arrows represent distance, thin blue curved arrows represent direction with respect to either an allocentric (θ) or egocentric (θ′) reference axis (thin blue straight lines). Other diagrammatic conventions are as in Fig. 1.

In African desert ants, PI may be the dominant navigation strategy [27]. In honeybees, vectorial information is transmitted through their remarkable dance language [28], [29], and used for relocating a goal [30]. In the arthropod navigation literature, PI is considered to be of such importance that there exists at least one canonical and one neural network model which uses each of the four standard classes of spatial coordinates i.e., egocentric Cartesian (EC), egocentric polar (EP), allocentric Cartesian (AC) and allocentric polar (AP) representations (Fig. 2). Yet numerous other models were published which did not seem to fit into any of these categories, motivating the development of a more general classification scheme [7].

To adequately cover all the existing equational and neural models of PI, the new classification scheme differentiated models on the basis of two independent properties of the spatial reference frame. The first property was whether the representation was centred on the animal (egocentric) or outside the animal (allocentric), consistent with existing literature. Note that for simplicity, it was previously assumed that most allocentric reference frames are also earth-centred (geocentric) which adequately described most arthropod experiments on PI [7]. However, in many rodent experiments, the allocentric reference frame is purposefully disengaged from the geocentric one. To be strictly correct, here we assume the “geocentric” descriptor refers to some, but not necessarily all, members of the superset of “allocentric” reference frames.

The second property used by the classification scheme was whether there was a need to update directional components during PI. If a position is represented along one or more predefined directions, like a Cartesian coordinate (Fig. 2C, 2D), then the representation was considered to be built from “static vectors” since the axes (vectors) have static directions. In contrast, if directions were variable and thus a change in position generally required a change in the direction component (such as polar coordinates – Fig. 2A, 2B), the representation was considered to be built from “dynamic vectors” since the axes (vectors) have dynamic directions. It has been noted previously [7] that the number of directions used in each model was not important. This means that a neural network consisting of three basis vectors behaves in essentially the same way as one consisting of hundreds of basis vectors. The results derived below do not require specification of the number of basis vectors. Hence the results are general for any model which fits under this classification scheme. The four extended classes, namely an *egocentric static vectorial representation* (ESVR), *egocentric dynamic vectorial representation* (EDVR), *allocentric static vectorial representation* (ASVR), and *allocentric dynamic vectorial representation* (ADVR) are summarized in Table 1, and discussed more extensively below (see also [7] for a detailed treatment).

**Table 1:** Extended classification of representations of Euclidean space.

Representation Class	Example	Mathematical Behaviour During PI	Tolerance to Noise
Allocentric dynamic vectorial representation (ADVR e.g. Fig. 2A, 3A)	Allocentric polar (AP)	IDW (ε)	Low
Egocentric dynamic vectorial representation (EDVR e.g. Fig. 2B, 3B)	Egocentric polar (EP)	IDW (ε−δ)	Low
Allocentric static vectorial representation (ASVR e.g. Fig. 2C, 3C)	Allocentric Cartesian (AC)	ADW	High
Egocentric static vectorial representation (ESVR e.g. Fig. 2D, 3D)	Egocentric Cartesian (EC)	IDW (−δ)	Low

### From Directed Walks to Path Integration

Theoretically, the process of PI continually adds measured displacements and supplies an animal with metric information about the overall distance and direction from home or some other location [7]. Such vectorial information may in principle be used in a variety of ways, including map construction, binding to place information, association with motor outputs, and to perform even more sophisticated tasks such as path planning. Indeed, an animal may store vectorial information about multiple important places in its world. However, in this work, we only consider the neural record of a single journey using PI under noisy conditions. We argue that task sophistication will generally lead to a greater sensitivity to PI error, not less. Therefore, if the simplest PI task is fundamentally impossible, then so are all ethologically relevant generalizations thereof. Discrete time models are used for ease of description of error terms, and for consistency with published theory on directed walks [20], [21].

In rodents, PI is currently thought to be an important mechanism which maintains the spatial consistency of place cell and grid cell firing fields [6], [9]–[13]. For example, the observation that such fields remain stable in darkness is generally interpreted as evidence that PI is used to maintain their spatial specificity, but non-visual localizing cues may contribute [31]. The corollary of this argument is that during PI, the neural network state can be decoded to calculate the animal's perceived current location [32]. In essence, our analysis is a theoretical comparison of the most accurate and precise neural record which could be obtained during noisy PI, with the actual path traversed by the hypothetical animal.

In order to apply the results of DW theory to PI, we will consider the exact opposite situation of the original DW model. Rather than intending to walk in a straight line but suffering from random physical perturbations during locomotion, we will model an animal which is walking in a perfectly straight line, but which updates the internal representation of its location using a noisy PI process fed by noisy sensory inputs, resulting in an internal DW occurring in representational space.

The displacement of the represented location associated with each step *n* of the internal DW will be denoted by Cartesian coordinates. This is the step displacement, not the final internal representation of position, which would be the sum total of all displacements 1 to *n* (e.g., see Text S1). Allocentric representations (whether static or dynamic vectorial) will be expressed as allocentric Cartesian coordinates, using the symbols *(U_n_, V_n_)*, whilst egocentric representations (static or dynamic vectorial) will be expressed as egocentric Cartesian coordinates, using the symbols *(U′_n_, V′_n_)*, thus making the results of PI using the four classes of spatial representation immediately comparable with the original DW theory. This procedure in no way alters the expected outcome, which depends purely on the actual representational class being used by the animal, nor does it imply the conclusions are limited to representations based on a single pair of Cartesian basis vectors.

**Figure 3:**
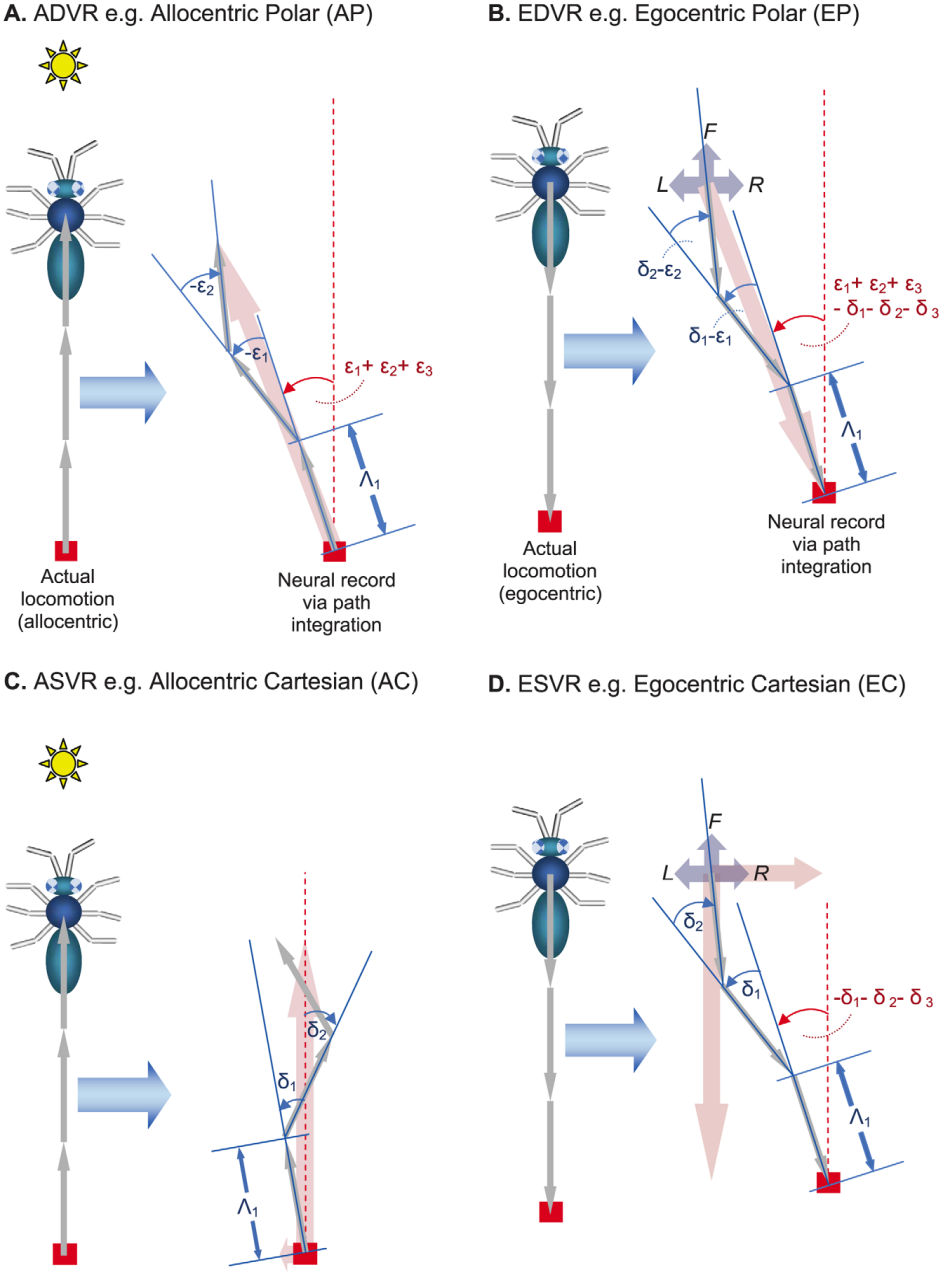
The effect of noise in different neural representations of space during PI. Note that each complete path is shown in representational space for clarity, but the process of PI only requires the maintenance of the current net position, ignoring previous steps. The examples shown are (**A**) allocentric dynamic vectorial representation (ADVR) e.g. allocentric polar (AP), (**B**) egocentric dynamic vectorial representation (EDVR) e.g. egocentric polar (EP), (**C**) allocentric static vectorial representation (ASVR) e.g. allocentric Cartesian (AC) and (**D**) egocentric static vectorial representation (ESVR) e.g. egocentric Cartesian (EC). Input rotational errors are denoted *δ*, update errors are denoted *ε*, and representational step lengths are denoted *Λ*. Actual locomotion is represented by three gray arrows in an allocentric (A and C) or egocentric (B and D) reference frame. The thick pink arrows represent distances, and egocentric forward (*F*), left (*L*) and right (*R*) are labelled for clarity. Other conventions are as in previous figures. See text for details.

Analogous to physical DWs, we model PI by assuming that at each step the animal intends to update its positional representation by a distance corresponding to the true step length (the scaling between physical and representational space is unimportant, and can be treated as unity) and in the direction corresponding to the true axis of physical locomotion (Figs. 3, Text S1). The actual representational step taken is of length 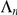, which deviates from the correct length by a random amount, corresponding to a failure of the animal to sense the true step length exactly. In all cases we consider the situation where allocentric directional information (e.g. a compass) is available every step, providing the measured allocentric heading, 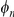, but with associated error term, *δ_n_*. This assumption was made since it has already been shown that in the absence of a compass, an IDW results irrespective of the navigation strategy [20], [21], and successful navigation is impossible (beyond a few steps).

For completeness, we take the spatial representation most tolerant to noise, and examine the effect of using purely idiothetic directional information i.e., only rotation measurement, 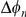, is available each step, and also associated with an equivalent *δ_n_* error term. Representational noise, *ε_n_*, is also assumed to corrupt the updating of the representation by the PI process each step. In the main text, only the update errors which determine ADW-like or IDW-like behaviour are discussed (but see Text S1).

Note that although it is convenient to use specific simulation examples for concept illustration, the theoretical results developed in this work are applicable to all animals which carry out PI, irrespective of the size of their nervous system, their evolutionary lineage or the known neurobiology.

## Results

We now show the equivalence between each of these four classes of spatial representation for PI and its corresponding directed walk, in the presence of neural noise. We show that any egocentric (ESVR or EDVR) or dynamic vectorial (ADVR or EDVR) representation of space accumulates noise in a way analogous to an IDW during PI. Therefore, PI using any such neural representation of space is inevitably associated with large random and systematic errors. In contrast, we show that an allocentric static vectorial representation (ASVR) accumulates noise in a way analogous to an ADW and therefore suffers from relatively small random errors and no systematic error during PI.

Formal proofs and stepwise geometric constructions showing the type and temporal order of error accrual are included in the supplement (Eqns S1.5–1.8 in Text S1, and Fig. S1). Here, we focus on key results which are necessary and sufficient to differentiate the performance of the four extended classes of spatial representations during PI. We use a theoretical construct, termed here a ‘neural record’, to illustrate the noise-induced divergence of the trajectory through representational space as indicated by the PI system, from the actual path of an animal. This hypothetical record is deduced from the changing internal states of the PI system during the journey, but calculated following the completion of a journey of *n* steps. For example, the neural record of step *m* is obtained by rearranging the vector equation following step *n*, giving current PI state in order to collect all the terms which should be associated with step *m*. Thus, for an egocentric and/or dynamic vectorial representation, the neural record is in fact the original step *m* corrupted by all subsequent errors up to and including those of step *n* (see below for analytical details). The neural record may therefore differ from the positions indicated by the set of initial PI states which resulted when each step was taken, but is a simple and intuitive way to track and visualize the errors arising during navigation.

### Egocentric Representations

Firstly we show that any egocentric spatial representation incorporates input error *δ* during PI in the same manner as an IDW, but in reverse temporal order.

An egocentric representation is one where places in the world are defined relative to the navigating agent. By definition, a right turn of a navigating agent implies the home direction has turned left by an equal magnitude. We know that using an egocentric representation, PI requires rotation (equivalent to a change in heading) as one input [7]. Due to the presence of biological noise, every step is associated with an angular error, *δ_n_*, in estimated heading rotation, irrespective of whether rotational signals are available directly e.g., 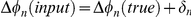, or if it is estimated from true compass bearings e.g., 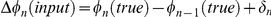.

An input rotational error of *δ_n_* results in a home direction error of *−δ_n_*. The result is that an error in current heading measurement is effectively added to all future steps in egocentric space (Figs. 3B, 3D, Fig. S1B, Fig. S1C). Consider a true trajectory which is perfectly straight in physical space such that true home is always directly behind the navigating animal. Following step one, any egocentric representation must incorporate the rotation error *−δ_1_*. We can trace the first step in representational space, which in egocentric Cartesian coordinates is 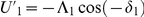 and 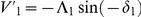 where *U′* represents the rostral-caudal (forward-backward) axis, and *V′* represents the lateral (left-right) axis (Fig 3D). For convenience, negative values of *U′* and *V′* mean backward and rightward respectively. The step length is denoted Λ in representational space. Analogous to the step length *L* in real space, Λ is assumed to incorporate some random error in representing the magnitude of forward displacement. In Fig. 3B (*δ* and *ε*) and Fig. 3D (*δ* only), we are considering the situation of an animal physically heading away from home, so by definition, the position of home moves in backward (−*U′*) direction in egocentric space whereas the position of the animal moves in a positive direction with respect to the home i.e., if viewed in an allocentric reference frame. During step two, an input rotation error of *δ_2_* results in a rotation of the entire current representation of home by *−δ_2_*. Hence following step two, both steps one and two have effectively incorporated the rotation *−δ_2_*, and so on (Fig. 3D shows pure *δ* accumulation). After *n* steps, the neural record of the *m*th step is given by
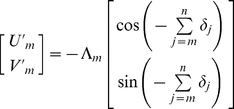
(3)It is important to note that the neural record of the *m*th step is dependent on the total number of steps, *n*, which has been taken. This is due to the fact that a rotational error resulting from each new step affects the entire PI record, which was built from all previous steps. Effectively, the neural record of step *m* is affected by 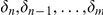 but not 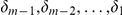. Thus, the angular error in representational space from step *m−1* to step *m* is 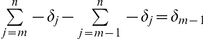 as illustrated in Fig. 3D. In an IDW, each new angular displacement 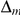 only affects step *m* and onwards, not steps *1* to *m−1*, which have already occurred [20]. However, in an egocentric spatial representation, an error at step *m* affects steps *1* to *m* in representational space i.e., those steps which have already been recorded. Careful analysis shows that the two representations can be considered as being equivalent. Without loss of generality we can renumber the steps in representational space in reverse order so that step *n* becomes step *1*, step *n−1* becomes step *2* and so on. Thus
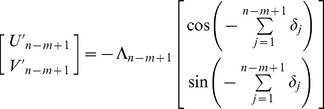
(4)which is mathematically equivalent to an idiothetic directed walk (IDW), but occurring in representational space (Eq. 2; Figs. 1B, 3B, 3D; Table S1; [22], [23]). Put simply, a straight trajectory in egocentric representational space accumulates angular errors in the reverse temporal order to an IDW in real space. Thus during an IDW in real space, recent rotational errors add to past ones, so earlier rotational errors contribute to all subsequent heading directions (Fig. 1B). In an egocentric representation, the most recent rotational input error, 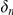, rotates the entire current neural representation of home (Fig. 3B, 3D), which consists of steps *1* to *n−1*, with their associated errors.

Note that the above arguments were developed independently of the type of egocentric representation. Therefore, the compass/rotation error *δ* is sufficient to cause a degradation of any egocentric representation of space so that a straight line in real space maps to an IDW in representational space (but see special case explained in Text S2). Thus an egocentric static vectorial representation (ESVR) or egocentric dynamic vectorial representation (EDVR; see Table 1) are both susceptible to the same type of path degenerescence in representational space. Of course, it is possible for other types of random errors to further degrade the egocentric representation (e.g. EDVR - see below).

### Dynamic Vectorial Representations

Next we show that a dynamic vectorial spatial representation incorporates update error *ε* during PI in the same manner as an IDW. A dynamic vectorial representation, typified by the polar representation, consists of vectors containing variable angular components. The path integrator's measure of direction accrues an error *ε* during the updating process, irrespective of the reference frame so 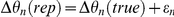 where 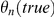 is the true current direction from home in an allocentric spatial representation. Unfortunately, the true net direction is not available, but only the approximation resulting from previous steps. The critical concept here is that the update error 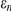 adds to the current net direction which was estimated from accumulating all previous steps i.e., 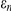 effectively rotates the representation of all previous steps. Using the same analysis conventions as the previous section, we examine the mapping of a straight trajectory into representation space using a dynamic vectorial representation. Following step one, an update error distorts the true value of *θ* or *θ′* by *ε*_1_ in representational space. In allocentric Cartesian coordinates (Fig. 3A), the straight trajectory in real space is aligned with the positive *U* axis so that 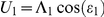 and 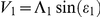.

Following step two, an allocentric dynamic vectorial representation accrues the update error *ε_2_*, which is effectively added to steps one and two, in a manner similar to rotation errors. After *n* steps, the neural record of the *m*th step is given by
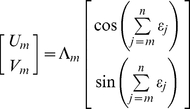
(5)Thus, the angular error in representational space from step *m−1* to step *m* is 
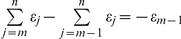
 as illustrated in Fig. 3A, Fig. S1A. In egocentric coordinates (Figs. 3B, Fig. S1C), the result of update errors is similar to the effect of rotation errors, except that the sign of the update error is preserved. Perhaps more importantly, as explained already, an egocentric representation is also affected by rotation errors. Thus following step one, 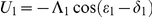, and 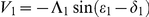. After *n* steps, the neural record of the *m*th step is given by
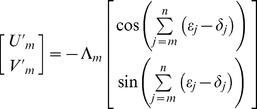
(6)Clearly, both types (allocentric or egocentric) of dynamic vectorial neural records are of the same mathematical form as the egocentric neural record. Therefore, the update error *ε* is sufficient to cause a degradation of any dynamic vectorial representation of space (ADVR or EDVR) so that a straight line in real space maps to an IDW in representational space.

### Allocentric Static Vectorial Representations

**Figure 4:**
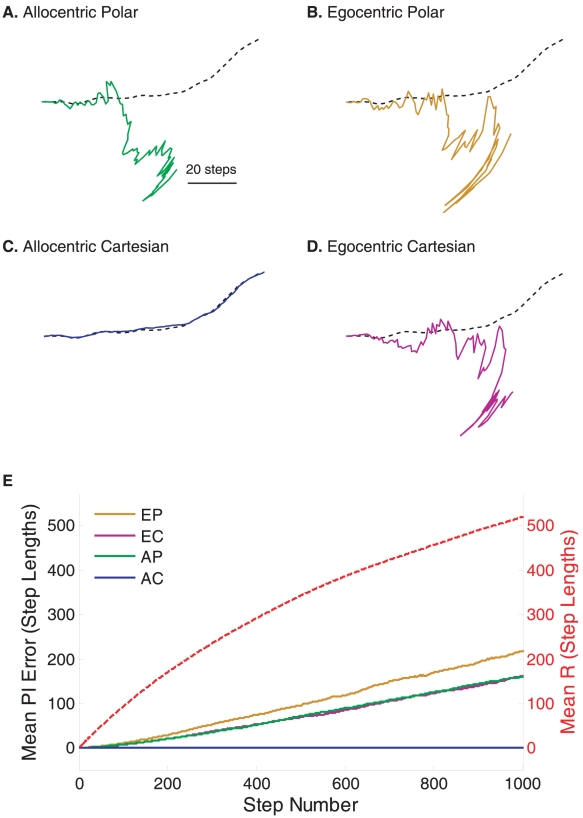
Quantifying the effect of noise during path integration (PI). An example is shown of noisy sensory inputs and home vector (HV) updating using allocentric polar (AP, **A**, green), egocentric polar (EP, **B**, gold), allocentric Cartesian (AC, **C**, blue), and egocentric Cartesian (EC, **D**, purple) coordinates. In each of the four examples, the actual path shown (dashed line) was an idiothetic directed walk (IDW) of 100 steps of step length 1 unit, generated assuming Gaussian random turns between successive steps, with a standard deviation of 0.1 radian. Gaussian noise with standard deviation of π/36 radians (5°) was added to compass readings and rotation measurements (denoted as *δ* in text). Noise during updating of HV coordinates was modelled by an independent Gaussian error ε, with standard deviation of π/36 units (equivalent to 5° for angular measurements). The trajectories traced by the HV in neural space are overlaid on the true path for the four coordinate systems. All simulations were based on exact discrete-time update equations (Table S2). (**E**) shows the average distance between the HV and the actual position from 1,000 simulated paths, extended to 1,000 steps. Note that the HV error function using AC coordinates (blue line) is very close to the abscissa. The mean radial distance from home, R, of the 1,000 IDWs are also shown (red dashed line). This sublinear relationship reflects cumulative heading rotations (intended or otherwise) of IDWs. The PI update equations used here are given in Table S2.

Now we show why an ASVR incorporates input and update errors (*δ* and *ε* respectively) during PI in the manner of an ADW. A static vectorial representation, typified by the Cartesian representation, consists of vectors containing fixed angular components. We know that using an allocentric representation, PI requires absolute heading as one input [*7*]. Since biological compasses are imperfect, there is an angular error *δ* associated with each step, much like the rotation error of egocentric representations. Thus, 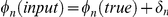. Again, we analyze the mapping of a straight trajectory in real space into representational space (Fig. 3C). Following step one, 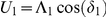 and 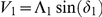. The neural record of the *m*th step is given by
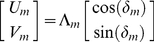
(7)which is mathematically equivalent to an allothetic directed walk (ADW), occurring in representational space (Fig. 1A, Fig. 3C; Table S1; [20], [21]). Update errors alone lead to a representational trajectory described by 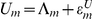 and 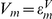, which is also equivalent to an ADW. Combining input and update errors,
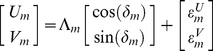
(8)which is also mathematically equivalent to an ADW. Therefore, in an allocentric static vectorial representation (ASVR; see Table 1), a straight line in real space maps to an ADW in representational space. Note that in all cases considered above, it was assumed that an allothetic directional cue was used as input. It is straightforward to show that using an idiothetic directional cue as input degrades performance further. Indeed, even for an ASVR, a straight line in real space maps to an IDW in representational space if only idiothetic directional cues are used i.e.,
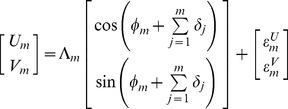
(9)The performances of the four classes of spatial representations were compared via computer simulation. An equational model from each class ([7], Table S2) was used to carry out PI using the same set of random trajectories (Fig. 4). For consistency all examples have directional/rotational input errors and update errors of equal magnitude. The neural record from one random example is shown (Fig. 4, A–D) overlaid on the true trajectory, scaled so that step length *L* = Λ.

The average positional estimation error (Fig. 4E) clearly demonstrates the superiority of the example from the ASVR class, consistent with theory. Variants of this class and limitations of errors are considered further below and in Text S3.

### Features of PI Models Using an ASVR

In the preceding analyses, the classification of spatial representations did not consider the modulus or length of the (static or dynamic) vectors used as the basis for a representation. The classification scheme used so far sufficed to give us the necessary insights into which types of spatial representations can map space faithfully via the process of PI. However, what does that tell us about the neural circuitry of navigation? Firstly, each class can be further subdivided to differentiate between fixed and variable length vectors [7].

**Figure 5:**
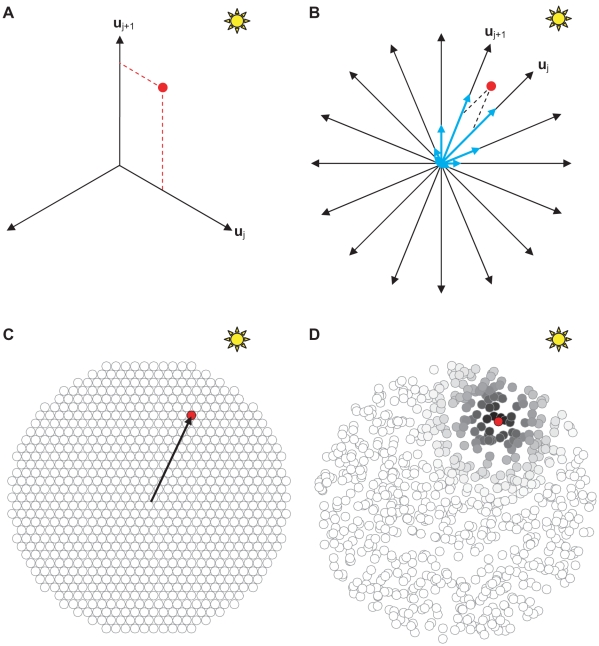
Theoretical variants of the allocentric static vectorial representation (ASVR) of 2-D Euclidean space. (**A**) is an ASVR example with 3 static vectors of dynamic moduli. A position (red dot) may be represented exactly by two scalar values (red dotted line projections) on the bounding static vectors **u**_j_ and **u**_j+1_ (see Text S2 for further details). (**B**) is an ASVR example with 16 static vectors of dynamic moduli. The scalar coordinates projected on adjacent basis vectors are shown as per (A). An approximate representation is also shown (blue arrows) where a position (or displacement) has a distributed representation. (**C**) shows a graphical example consisting of static vectors (ends shown as circles) with static moduli, distributed in a closest packing arrangement. Each position (e.g. red dot) is designated by one particular static vector with a binary response to indicate the navigating agent's presence or absence at that position. Note that the start location during PI is arbitrary from a computational perspective, but once set (e.g. start of arrow) it is expected to remain stable for at least the duration of the current journey. (**D**) shows a graphical example consisting of static vectors with static moduli, distributed randomly. Here the greyscale shading indicates a graded response which may be inversely related to the proximity of the agent's position (red dot) to each static vector's optimally tuned position, or be a likelihood estimate of the agent being at any particular position.

By variable length static vectors we mean that the representation is based on vectors defining fixed directions (such as X and Y axes), but not fixed distances in these directions i.e., basis vectors. The representation records the (variable) distance moved along these fixed directions. Figure panels 5A and 5B show two graphical examples of a variable length SVR system used to represent the same net allocentric displacement (red disc). In Fig. 5A, there are three static basis vectors and a simple, mathematically exact, decomposition of the red disc's components is shown. In Fig. 5B, an ASVR with many basis vectors is shown, along with the components of the red disc, but also an example of a response function (analogous to distributing vector components) of the same displacement. A range of PI models have been published, particularly in the arthropod literature, which fall into this subclass [7]. The neural model implementations of an ASVR (dynamic moduli) are often drawn as a ring-like array of neurons, with each neuron representing a fixed allocentric direction, with receptive fields of various widths and shapes. Of course, the ring configuration could be an artefact of the compass input needed for an allocentric representation. Nonetheless, the computational requirement of a direct correspondence between allocentric angular space and neuron index is suggestive of a structured organization.

In contrast, a set of static vectors with a range of fixed lengths, spread out over the 2-D Euclidean space is reminiscent of a grid or map. Effectively, each vector represents a point, or small region, in space. Then, the representation of a position no longer requires spatial measurements like length or angle. At its simplest, a binary output suffices, which denotes a location is either at a static vector or not (Fig. 5C). More information may be represented by a distribution of output values corresponding to probability, which could account for positional uncertainty (e.g. Fig. 5D).

The intuitive division of allocentric static vectors into fixed and variable lengths naturally produces models which resemble place cell maps and neural rings, respectively. Interestingly, published arthropod neural models of navigation typically adopt a ring like structure even those which are now known to be noise-intolerant, with one notable exception [33]. In contrast, mammalian neural models have typically been based on map-like networks [9]–[11], [34]. Whether this is a coincidence or reflects fundamental biology remains unclear. Some important neural architectural and computational issues of the two ASVR subclasses are considered below.

## Discussion

### The Error of Our Ways

Four general classes of spatial representations were studied. The motivations for using this scheme were twofold. Firstly, standard classification systems have been insufficient to account for a number of neural network models. Secondly, the general classification scheme is consistent with mathematical results from DW theory which proved that the critical determinant of trajectory behaviour is whether angular errors accumulate. In particular, there is no dependence on the number of axes used to represent a position or whether linear errors accumulate. For instance, ring-like and map-like neural structures may be considered alongside simpler counterparts, even equational models.

### Noisy Walks in Noisy Brains

From first principles, we showed how real space is mapped into different classes of representational space via PI in the presence of noise. It was found that real navigation journeys represented in allocentric dynamic vectorial representations (ADVRs), egocentric dynamic vectorial representations (EDVRs), or egocentric static vectorial representations (ESVRs) are corrupted by noise in similar ways. Examples include all egocentric (e.g. EC or EP) and all polar (e.g. AP or EP) representations. A straight trajectory in real space maps to an IDW in representational space, resulting in nonlinear systematic errors and irrecoverably large random errors. Consequently, the error of spatial representation is expected to increase rapidly, rendering the animal hopelessly lost. Egocentric representations suffer particularly from input noise, *δ*, while dynamic vectorial representations are particularly affected by update noise *ε*. In this work, the magnitude of noise was not considered – only that noise exists. Although the properties of biological sensor noise may be well characterized in certain cases [35], neural processing noise is typically much more difficult to quantify. It is possible for instance that input noise is much larger in magnitude than update noise. Our results then predict ESVRs and EDVRs to show greater nonlinear systematic errors than ADVRs. However, due to the nature of IDWs, the random errors of ADVRs would eventually exceed those of ESVRs or EDVRs, thereby causing even greater PI inaccuracies, albeit delayed.

In contrast, allocentric static vectorial representations (ASVRs), typified by the allocentric Cartesian (AC) representation, faithfully capture the geometric and metric properties of real trajectories. A straight trajectory in real space maps to an ADW in representational space. In principle, animals which have evolved ASVRs would have far superior navigational outcomes, particularly for long journeys. For theoretical completeness, we note that ASVRs are not entirely immune to neural noise. For instance, large systematic angular errors (e.g. >90°) can still cause failure of homing via PI using a compass plus an ASVR (Text S3). However, we believe that such extreme errors are unlikely to occur in nature, and in any case would cause even more severe problems for alternative neural representations of space.

The strengths and weaknesses of existing arguments for or against using different representation systems to model arthropod PI have been reviewed [7], [36], [37]. Can the modelling literature say anything about the current results? In fact, under noisy conditions, and using evolutionary algorithms to optimize performance, the evolved PI neural networks were found to be subtypes of ASVRs [38], [39]. This is entirely consistent with the current theoretical results. Admittedly, the published models made *a priori* assumptions which might have unfairly favoured an ASVR.

As noted previously, most models neglected the effects of noise. Even in the absence of noise, a number of computational properties discourage the use of non-ASVRs. These include large rates of change in angle needed for ADVRs and EDVRs near home, large rates of change in position needed for ESVRs far from home, feedback of current path integrator state into the update process for all non-ASVRs, among others [7]. Individually, the arguments made assuming noise-free conditions could be countered. Nevertheless, the weight of evidence seemed to favour ASVRs. In combination with the clear consequences of neural noise, the case for ASVRs is difficult to dispute. In light of this, new interpretations of previous experimental results [e.g. 40] may be required, and assumptions of non-ASVR systems for biological PI [e.g. 41] should be re-examined.

### Rings and Maps – Tools of an Adventurer

At least two subclasses of ASVR exist, which for convenience can be approximately described as ring-like (Fig. 5A, 5B) and map-like representations of space (Fig. 5C, 5D). While there does not appear to be a significant difference in noise-tolerance between the two ASVR subclasses, there may be distinctions based on phylogeny or behavioural requirements.

In the literature, there appears to be a lack of map-like models of arthropod PI, and a lack of ring-like models of mammalian PI. Interestingly, arthropod PI models, including the noise-tolerant ring-like varieties, were inspired by behavioural results. This suggests ring-like models are well suited to account for a variety of navigation behaviours related to PI [7]. In contrast, models of mammalian PI were inspired by *in vivo* recordings. In other words, neurons are known to exist which possess the necessary properties to represent space in a noise-tolerant way. It is tempting to hypothesize that this apparent dichotomy in published models reflects a fundamental difference between the nervous systems of arthropods and mammals. Unfortunately, evidence is lacking. Nonetheless, our results provide strong theoretical justification for the evolution of some sort of ASVR, in any species which needs to navigate or represent space. Differentiating between the two subclasses of ASVR, however, may present significant theoretical and experimental challenges and is the subject of ongoing research. Some important neural architectural and computational issues are briefly outlined below.

It is relatively simple to envisage a direct correspondence between allocentric angular space and a neuronal array, particularly when a compass is available. This is analogous to the ring-like subclass of ASVR. Unfortunately, there is a lack of electrophysiological evidence for any particular type of PI system in arthropods. From the tenuous data, a possible candidate for a ring-like PI system may be the central complex [15].

For the map-like ASVR subclass, it is positional space which corresponds to a neuronal array - but this is not trivial to achieve. For instance, one might assume that using allocentric landmarks allows for relatively precise spatial localization, in the same way a compass allows for angular localization. It might be further assumed that a map-like spatial representation can therefore be generated using allocentrically-stable cues. Yet the association of landmarks to a particular spatial location requires visiting that location – but how could that location be encoded in the first place? What determines the spatial relationship to other positions?

One possibility is that the spatial representation is generated dynamically, during the first visit to any physical location, with the recruitment of neural units *en route*. However, this seems unlikely since it results in a circular argument i.e., PI using a map-like ASVR requires a pre-existing “map”, which is not present until the path is recorded via PI. Although landmarks are excellent for localization of individual places in the world, they are generally poor for relating those places in a metrically consistent way (unless the spatial layout of the landmarks are already known). Hence it is likely that a pre-existing spatial representation is used during PI rather than being formed dynamically.

It is worth noting that many SLAM (Simultaneous Localization and Mapping) algorithms have been implemented successfully in engineering and robotic applications [42], and superficially appear to contradict this assertion. However, careful examination of the algorithms reveals that in fact there is always a predefined representation of space, often metric and Cartesian-based, but empty to begin with (and without necessarily pre-allocating much memory resource). The SLAM algorithms serve to bind those spatial representations with objects and experiences during navigation. Even here, the spatial representation cannot be formed *ab nihilo* on encountering a landmark, but must be generated *en route* to maintain a consistent spatial relationship or spatial metric.

If a map-like ASVR cannot dynamically bind physical space (and the corresponding allocentric sensory information) to neural substrates, then how can PI be achieved? An alternative model might involve pre-existing, metric relationships between all the neurons in an array – literally a “place map”. In this way, PI can be achieved by translation of an activity bump, for instance, along a network of neurons in a spatially consistent way [e.g. 9], [10]. Most mammalian PI models have been developed to explain *in vivo* data parsimoniously and are typically of the map-like ASVR subclass. However, from a computational standpoint further analysis is required to determine whether modified ring-like ASVR models may also explain *in vivo* data. Of note is the fact that the electrophysiological properties of place cells and grid cells are dynamically affected by allocentric cues, unlike simple place map models. Multiple ring-like ASVR systems may allow remapping to occur readily, yet maintain a consistent metric relationship between real places in representational space.

Behavioural data may also offer clues for differentiating between variable and fixed length ASVRs. Ring-like models have been used with some success to model systematic errors of PI. Can models using map-like representations do the same? Searching behaviour is often associated with PI, particularly following displacement experiments. Interestingly, search behaviour seems to reflect both the accrued uncertainty of the outbound journey, as well as the dynamically changing prior and posterior probability distributions during searching [43]–[45]. Can ring-like models maintain sufficient information to account for such complexities? Rigorous theoretical analyses of these issues may yield further insights about the neural representation of space and is currently under way.

### Conclusions

PI is an ancient and ubiquitous navigation strategy. Even highly complex animals such as rodents and humans possess a PI system. Due to the ubiquitous presence of biological noise, a variant of an ASVR is most likely to be used. If other navigation strategies, and indeed other neural functions, evolved from a PI ancestry, there are likely to be residual ASVR signatures in modern nervous systems. Their properties remain an open topic for future investigation.

The current work advances our understanding of PI, animal navigation, evolved neural systems, and further demonstrates the usefulness of DW theory. These analytical foundations will hopefully steer future experimentation, and focus modelling work, towards a deeper understanding of biological navigation and animal nervous systems. For example, a biological implementation of a ring-like ASVR model might entail neurons which are tuned to specific allocentric directions, and which behave like odometers in their preferred directions. If such an odometer is linear with respect to distance, it might be expected that PI fails catastrophically beyond a certain radial distance from home, once some ceiling value is reached. Alternatively, if distance is encoded in a saturating manner to avoid a ceiling range, the inherent uncertainty in the representation of space may increase nonlinearly with distance, which may manifest in the size of searching distributions following PI. In map-like ASVR representations, there is a need for translating the current position during PI. Is that achieved via interneurons perhaps like an attractor network? Do all interneurons receive the same allocentric heading signal? Does the map have an edge or does the map wrap around seamlessly like a torus? How is positional uncertainty represented?

We believe the results presented in this work represent the strongest theoretical foundation to date for determining the type of spatial representation likely to be used by biological nervous systems for navigation.

## Supporting Information

Figure S1 Stepwise geometric constructions of cumulative errors in representational space during path integration (PI).(A) In an allocentric framework, using an allocentric polar (AP) representation of space during PI results in an accumulation of some amount of update error, ε, during each step. Both clockwise (CW) and counterclockwise (CCW) update errors are illustrated. (B) In an egocentric framework, using an egocentric Cartesian (EC) representation of space during PI results in an accumulation of some amount of input error, δ, during each step. An angular error (−δ) in the compass (sun symbol) reading gives rise to an inferred rotation (input to PI system) in the opposite direction (δ), which results in a home vector shifted by −δ in representational space. The thick arrows denote forward, leftward and rightward. (C) In an egocentric framework, using an egocentric polar (EP) representation of space during PI results in an accumulation of some amount of update error, ε, in addition to some amount of input error, δ, during each step. Note that the sign of the update error, ε, is independent of the spatial framework used (e.g., a CW rotation is CW in both egocentric and allocentric reference frames).
(0.24 MB EPS)

Table S1 Parametric error accumulation equations describing trajectories in 2D real and representational space for directed walks consisting of general elementary steps.
(0.10 MB DOC)

Table S2 Discrete-time HV update equations used for simulations.
(0.05 MB DOC)

Text S1 General properties of real and representational trajectories.
(0.13 MB DOC)

Text S2 A special case: using compass readings to estimate rotations.
(0.06 MB DOC)

Text S3 Allocentric static vectorial representations.
(0.14 MB DOC)
