# Supplementary material for: Finding the Way with a Noisy Brain
Source: PLoS Comput Biol. 2010 Nov 11;6(11):e1000992. doi: 10.1371/journal.pcbi.1000992 (PMC2978673; doi:10.1371/journal.pcbi.1000992)
Supplement: Table S1 — Parametric error accumulation equations describing trajectories in 2D real and representational space for directed walks consisting of general elementary steps. (0.10 MB DOC) [file pcbi.1000992.s002.doc]

**Table S1 – Parametric error accumulation equations describing trajectories in 2D real and representational space for directed walks consisting of general elementary steps.** Equations show the displacement associated with any step *m* after a total of *n* steps have been taken (i.e. $1\leq m\leq n$). Superscripts ^a^ and ^b^ refer to errors associated with the first and second angular components of a general elementary step respectively (see [*21*] for details).

| Type of Directed Walk | Representational displacement^†^ | Real displacement^†^ |
| --- | --- | --- |
| ADW | $U_{m}=\Lambda_{m}$ $V_{m}=0$ | $X_{m}=L_{m}\cos\left(\Delta_{m}^{a}+\Delta_{bias}^{a}\right)$ $Y_{m}=L_{m}\sin\left(\Delta_{m}^{a}+\Delta_{bias}^{a}\right)$ |
| IDW | $U_{m}=\Lambda_{m}$ $V_{m}=0$ | $X_{m}=L_{m}\cos\left(\sum_{j=1}^{m} \left(\Delta_{j}^{a}+\Delta_{bias}^{a}\right)+\sum_{j=1}^{m-1} \left(\Delta_{j}^{b}+\Delta_{bias}^{b}\right)\right)$ $Y_{m}=L_{m}\sin\left(\sum_{j=1}^{m} \left(\Delta_{j}^{a}+\Delta_{bias}^{a}\right)+\sum_{j=1}^{m-1} \left(\Delta_{j}^{b}+\Delta_{bias}^{b}\right)\right)$ |
| Type of Spatial Representation | Real displacement^†^ | Representational displacement^‡^ |
| ASVR | $X_{m}=L_{m}$ $Y_{m}=0$ | $U_{m}=\Lambda_{m}\cos\left(\delta_{m}^{a}+\delta_{bias}^{a}\right)+\varepsilon_{m}^{U}$ $V_{m}=\Lambda_{m}\sin\left(\delta_{m}^{a}+\delta_{bias}^{a}\right)+\varepsilon_{m}^{V}$ |
| ADVR | $X_{m}=L_{m}$ $Y_{m}=0$ | $U_{m}=\Lambda_{m}\cos\left(\delta_{m}^{a}+\delta_{bias}^{a}+\sum_{j=m}^{n} \varepsilon_{j}^{\theta}\right)$ $V_{m}=\Lambda_{m}\sin\left(\delta_{m}^{a}+\delta_{bias}^{a}+\sum_{j=m}^{n} \varepsilon_{j}^{\theta}\right)$ |
| ESVR | $X_{m}=L_{m}$ $Y_{m}=0$ | $U'_{m}=-\Lambda_{m}\cos\left(-\sum_{j=m}^{n} \left(\delta_{j}^{a}+\delta_{bias}^{a}+\delta_{j}^{b}+\delta_{bias}^{b}\right)\right)+\varepsilon_{m}^{U'}$ $V'_{m}=-\Lambda_{m}\sin\left(-\sum_{j=m}^{n} \left(\delta_{j}^{a}+\delta_{bias}^{a}+\delta_{j}^{b}+\delta_{bias}^{b}\right)\right)+\varepsilon_{m}^{V'}$ |
| EDVR | $X_{m}=L_{m}$ $Y_{m}=0$ | $U'_{m}=-\Lambda_{m}\cos\left(\sum_{j=m}^{n} \left(\varepsilon_{j}^{\theta'}-\delta_{j}^{a}-\delta_{bias}^{a}-\delta_{j}^{b}-\delta_{bias}^{b}\right)\right)$ $V'_{m}=-\Lambda_{m}\sin\left(\sum_{j=m}^{n} \left(\varepsilon_{j}^{\theta'}-\delta_{j}^{a}-\delta_{bias}^{a}-\delta_{j}^{b}-\delta_{bias}^{b}\right)\right)$ |

^†^In allocentric Cartesian coordinates.

^‡^In allocentric or egocentric Cartesian coordinates according to spatial representation.
