# Supplementary material for: Finding the Way with a Noisy Brain
Source: PLoS Comput Biol. 2010 Nov 11;6(11):e1000992. doi: 10.1371/journal.pcbi.1000992 (PMC2978673; doi:10.1371/journal.pcbi.1000992)
Supplement: Table S2 — Discrete-time HV update equations used for simulations. (0.05 MB DOC) [file pcbi.1000992.s003.doc]

**Table S2 - Discrete-time HV update equations used for simulations** (see Fig. 4)

| Coordinate system | HV^†^ update equations |
| --- | --- |
| Allocentric Cartesian |  |
| Allocentric Polar |  |
| Egocentric Cartesian |  |
| Egocentric Polar |  |

^†^The HV (home vector) is a term used commonly in the path integration literature to describe the distance and direction of ‘home’ which is estimated and continually updated by the path integration apparatus. However, allocentric representations actually maintain a net displacement from the starting position. Homing requires a transformation of the coordinates [7]. Furthermore, path integrator states may be associated with goals other than the home.

Note that the equations listed here are state update equations for specific implementations of PI, using specific inputs, and left in their native frame of reference. Hence they are distinct from the stepwise displacement equations used elsewhere in this work, which have been mapped into egocentric or allocentric Cartesian coordinates to aid comparison with directed walks.
