# Supplementary material for: Finding the Way with a Noisy Brain
Source: PLoS Comput Biol. 2010 Nov 11;6(11):e1000992. doi: 10.1371/journal.pcbi.1000992 (PMC2978673; doi:10.1371/journal.pcbi.1000992)
Supplement: Text S1 — General properties of real and representational trajectories. (0.13 MB DOC) [file pcbi.1000992.s004.doc]

**S1 General Properties of Real and Representational Trajectories**

In the majority of this work, for clarity we deal with directed walks consisting of simple elementary steps [20]. It should be obvious to the reader that extension to the more comprehensive general elementary step [21] follows the same principles. Hence the results presented are in fact independent of error distribution, presence of biases or statistical interdependence of error components [21]. For completeness, Table S1 outlines the application of DWs using general elementary steps to the noisy neural representations.

We begin with two conceptually distinct constructs (see main text for more descriptive details) i.e.,

1. A straight trajectory in representational space realized using a compass (allothetic directed walk - ADW) or not using a compass (idiothetic directed walk - IDW)
2. A straight trajectory in real space recorded by 4 different classes of spatial representations (allocentric static vectorial representation - ASVR, allocentric dynamic vectorial representation - ADVR, egocentric static vectorial representation - ESVR, egocentric dynamic vectorial representation - EDVR)

Consistent with published results, we designate the X-axis (real space) to be the axis of intended locomotion. The basic mathematical construct of directed walks involves a straight trajectory in representational space which, due to unavoidable sensorimotor noise, results in an ADW or IDW in real space depending on the type of directional cue used (Fig. 2; Table S1). In an analogous manner, we consider a straight trajectory in real space which, due to unavoidable neural update noise, results in an ADW or IDW in representational space depending on the type of spatial representation used (Fig. 3; Table S1).

From careful analysis and aid of geometric constructions (Figs. 2-3), we can write down the displacement of the *m*’th step in real (*X_m_*, *Y_m_*) and either allocentric representational (*U_m_*, *V_m_*) space, or egocentric representational (*U’_m_*, *V’_m_*) space. Note that we are using Cartesian coordinates to describe the representational trajectory, for consistency with the conventions of directed walk theory [20, 21] and for its general intuitive appeal. It should be noted that this does not mean the neural representation of space is using Cartesian coordinates but rather emphasizes the utility of coordinate transforms for trajectory analyses [7]. Fig. S1 shows three examples of stepwise accumulation of input and update errors during PI. This is a more detailed, step-by-step geometric constructions showing the important cumulative errors summarized in Fig. 3 (panels A, D and B respectively). An alternative analytical description of ADWs, IDWs, ASVRs, ADVRs, ESVRs, and EDVRs using recurrent equations is presented below.

We demonstrate formally using recurrent equations the equivalence between directed walks (ADWs and IDWs) and the result of using PI in the four extended classes of spatial representations (ASVR, ADVR, ESVR and EDVR). Briefly, the logic can be summarized as follows. A vector difference equation defines the iterative PI update procedure: taking the previous PI state-vector following step *j-1*, add the current step using the estimated distance ($\Lambda_{j}$) and direction ($\delta_{j}$), then apply the update error (which varies with the representational class) to give the current PI state-vector following step *j*. The difference equations are then used to derive the PI state after *n* steps as a function of all past steps (see S1.5-S1.8, bottom lines). For egocentric and dynamic vectorial representations, the equations show us that the contribution (or *neural record*) of a past step *j* acting on the present state *n* is always the original step (distance $\Lambda_{j}$, direction $\delta_{j}$) subjected to all subsequent errors *j* to *n*. This shows the *neural records* of past steps to be like the physical steps of an IDW (S1.4) but in reverse temporal sequence, since an IDW step is affected by all previous (rather than subsequent) errors.

For the formal analysis, we define the rotation matrix

$\mathbf{R}\left(\omega\right)=\left[\begin{matrix} \cos\omega& -\sin\omega\\ \sin\omega& \cos\omega\end{matrix}\right]$ (S1.1)

and note that $\mathbf{R}\left(\omega_{1}\right)\times\mathbf{R}\left(\omega_{2}\right)=\mathbf{R}\left(\omega_{1}+\omega_{2}\right)$. Extending on the earlier definition of displacements in a neural representation of space, the net displacement vector in an allocentric Cartesian reference frame is

$\left[\begin{matrix} U_{n} \\ V_{n} \end{matrix}\right]_{Total}=\sum_{j=1}^{n} \left[\begin{matrix} U_{j} \\ V_{j} \end{matrix}\right]$ (S1.2)

During an ADW, the navigating agent makes an unavoidable angular displacement error Δ during each step (of length L). However, it is assumed to have access to an allothetic directional cue which provides absolute bearing i.e., a compass. It is then assumed to fully correct its heading error, prior to taking the next step, during which it makes another unavoidable angular displacement error and so forth (see [20, 21] for more detail). Using recurrent equations, the total displacement following *n* steps can be expressed as follows:

$\begin{matrix} \left[\begin{matrix} X_{n} \\ Y_{n} \end{matrix}\right]_{Total}=\left[\begin{matrix} X_{n-1} \\ Y_{n-1} \end{matrix}\right]_{Total}+\mathbf{R}\left(\Delta_{n}\right)\left[\begin{matrix} L_{n} \\ 0 \end{matrix}\right] \\ =\left[\begin{matrix} X_{n-2} \\ Y_{n-2} \end{matrix}\right]_{Total}+\mathbf{R}\left(\Delta_{n-1}\right)\left[\begin{matrix} L_{n-1} \\ 0 \end{matrix}\right]+\mathbf{R}\left(\Delta_{n}\right)\left[\begin{matrix} L_{n} \\ 0 \end{matrix}\right] \\ =\cdots=\sum_{j=1}^{n} \mathbf{R}\left(\Delta_{j}\right)\left[\begin{matrix} L_{j} \\ 0 \end{matrix}\right] \end{matrix}$ (S1.3)

This is the realized displacement assuming the original intended trajectory was along a strength line (arbitrarily designate as the X-axis).

During an IDW, the navigating agent has available idiothetic cues only (no compass) and therefore is unable to correct its heading following each step. Consequently, angular displacement errors (Δ s) accumulate from step to step. Using recurrent equations, the total displacement following *n* steps can be expressed as follows:

$\begin{matrix} \left[\begin{matrix} X_{n} \\ Y_{n} \end{matrix}\right]_{Total}=\left[\begin{matrix} X_{n-1} \\ Y_{n-1} \end{matrix}\right]_{Total}+\prod_{i=1}^{n} \mathbf{R}\left(\Delta_{i}\right)\left[\begin{matrix} L_{n} \\ 0 \end{matrix}\right] \\ =\left[\begin{matrix} X_{n-2} \\ Y_{n-2} \end{matrix}\right]_{Total}+\prod_{i=1}^{n-1} \mathbf{R}\left(\Delta_{i}\right)\left[\begin{matrix} L_{n-1} \\ 0 \end{matrix}\right]+\prod_{i=1}^{n} \mathbf{R}\left(\Delta_{i}\right)\left[\begin{matrix} L_{n} \\ 0 \end{matrix}\right] \\ =\cdots=\sum_{j=1}^{n} \left(\prod_{i=1}^{j} \mathbf{R}\left(\Delta_{i}\right)\left[\begin{matrix} L_{j} \\ 0 \end{matrix}\right]\right) \\ =\sum_{j=1}^{n} \mathbf{R}\left(\sum_{i=1}^{j} \Delta_{i}\right)\left[\begin{matrix} L_{j} \\ 0 \end{matrix}\right] \end{matrix}$ (S1.4)

We now examine the various classes of spatial representation which may be used for path integration (PI) – see main text for further details. In an allocentric Cartesian reference frame, using an ASVR,

$\begin{matrix} \left[\begin{matrix} U_{n} \\ V_{n} \end{matrix}\right]_{Total}=\left[\begin{matrix} U_{n-1} \\ V_{n-1} \end{matrix}\right]_{Total}+\mathbf{R}\left(\delta_{n}\right)\left[\begin{matrix} \Lambda_{n} \\ 0 \end{matrix}\right]+\left[\begin{matrix} \varepsilon_{n}^{U} \\ \varepsilon_{n}^{V} \end{matrix}\right] \\ =\left[\begin{matrix} U_{n-2} \\ V_{n-2} \end{matrix}\right]_{Total}+\mathbf{R}\left(\delta_{n-1}\right)\left[\begin{matrix} \Lambda_{n-1} \\ 0 \end{matrix}\right]+\mathbf{R}\left(\delta_{n}\right)\left[\begin{matrix} \Lambda_{n} \\ 0 \end{matrix}\right]+\left[\begin{matrix} \varepsilon_{n-1}^{U} \\ \varepsilon_{n-1}^{V} \end{matrix}\right]+\left[\begin{matrix} \varepsilon_{n}^{U} \\ \varepsilon_{n}^{V} \end{matrix}\right] \\ =\cdots=\sum_{j=1}^{n} \mathbf{R}\left(\delta_{j}\right)\left[\begin{matrix} \Lambda_{j} \\ 0 \end{matrix}\right]+\sum_{j=1}^{n} \left[\begin{matrix} \varepsilon_{j}^{U} \\ \varepsilon_{j}^{V} \end{matrix}\right] \end{matrix}$ (S1.5)

The sum which involves the displacement in representational space, Λ, is analogous to the trajectory of an ADW in real space. Note that for unbiased update errors, $\left\langle\varepsilon\right\rangle=0$, which do not affect the average neural record during PI. They do, however, contribute to the overall positional uncertainty. Hence these are effectively random errors superimposed on the positional uncertainty of an ADW.

Using an ADVR (e.g. Fig. S1A),

$\begin{matrix} \left[\begin{matrix} U_{n} \\ V_{n} \end{matrix}\right]_{Total}=\mathbf{R}\left(\varepsilon_{n}^{\theta}\right)\left(\left[\begin{matrix} U_{n-1} \\ V_{n-1} \end{matrix}\right]_{Total}+\mathbf{R}\left(\delta_{n}\right)\left[\begin{matrix} \Lambda_{n} \\ 0 \end{matrix}\right]\right) \\ =\mathbf{R}\left(\varepsilon_{n}^{\theta}\right)\left(\mathbf{R}\left(\varepsilon_{n-1}^{\theta}\right)\left(\left[\begin{matrix} U_{n-2} \\ V_{n-2} \end{matrix}\right]_{Total}+\mathbf{R}\left(\delta_{n-1}\right)\left[\begin{matrix} \Lambda_{n-1} \\ 0 \end{matrix}\right]\right)+\mathbf{R}\left(\delta_{n}\right)\left[\begin{matrix} \Lambda_{n} \\ 0 \end{matrix}\right]\right) \\ =\cdots=\sum_{j=1}^{n} \mathbf{R}\left(\delta_{j}+\sum_{i=j}^{n} \varepsilon_{i}^{\theta}\right)\left[\begin{matrix} \Lambda_{j} \\ 0 \end{matrix}\right] \end{matrix}$ (S1.6)

This is consistent with the form of an IDW, with the exception of a single rotational input error, *δ*, which adds further noise to the neural representation. Note that not all ADVRs have a linear (modulus) component as polar representations do. Therefore, we do not specifically include an update error for this component. However, were it present, it would add noise to both egocentric axes, further corrupting the neural PI record.

Using an ESVR (e.g. Fig. S1B),

$\begin{matrix} \left[\begin{matrix} U'_{n} \\ V'_{n} \end{matrix}\right]_{Total}=\mathbf{R}\left(-\delta_{n}\right)\left(\left[\begin{matrix} U'_{n-1} \\ V'_{n-1} \end{matrix}\right]_{Total}+\left[\begin{matrix} -\Lambda_{n} \\ 0 \end{matrix}\right]\right)+\left[\begin{matrix} \varepsilon_{n}^{U'} \\ \varepsilon_{n}^{V'} \end{matrix}\right] \\ =\mathbf{R}\left(-\delta_{n}\right)\left(\mathbf{R}\left(-\delta_{n-1}\right)\left(\left[\begin{matrix} U'_{n-2} \\ V'_{n-2} \end{matrix}\right]_{Total}+\left[\begin{matrix} -\Lambda_{n-1} \\ 0 \end{matrix}\right]\right)+\left[\begin{matrix} \varepsilon_{n-1}^{U'} \\ \varepsilon_{n-1}^{V'} \end{matrix}\right]+\left[\begin{matrix} -\Lambda_{n} \\ 0 \end{matrix}\right]\right)+\left[\begin{matrix} \varepsilon_{n}^{U'} \\ \varepsilon_{n}^{V'} \end{matrix}\right] \\ =\cdots=-\sum_{j=1}^{n} \mathbf{R}\left(-\sum_{i=j}^{n} \delta_{i}\right)\left[\begin{matrix} \Lambda_{j} \\ 0 \end{matrix}\right]+\sum_{j=1}^{n-1} \mathbf{R}\left(-\sum_{i=j+1}^{n} \delta_{i}\right)\left[\begin{matrix} \varepsilon_{j}^{U'} \\ \varepsilon_{j}^{V'} \end{matrix}\right]+\left[\begin{matrix} \varepsilon_{n}^{U'} \\ \varepsilon_{n}^{V'} \end{matrix}\right] \end{matrix}$ (S1.7)

The first sum is analogous to an IDW (with net backward displacement in an egocentric reference frame), whereas the latter terms contribute to the positional variance but not the expected position, much like the update errors in an ASVR. For further clarity (and consistency with explanations in the main text), the step indices may be relabelled. Following *n* steps, the representational displacement attributed to step *m* is

$\left[\begin{matrix} U'_{m} \\ V'_{m} \end{matrix}\right]=-\mathbf{R}\left(-\sum_{i=m}^{n} \delta_{i}\right)\left[\begin{matrix} \Lambda_{m} \\ 0 \end{matrix}\right]+\mathbf{R}\left(-\sum_{i=m+1}^{n} \delta_{i}\right)\left[\begin{matrix} \varepsilon_{m}^{U'} \\ \varepsilon_{m}^{V'} \end{matrix}\right]+\left[\begin{matrix} \varepsilon_{n}^{U'} \\ \varepsilon_{n}^{V'} \end{matrix}\right]$ (S1.7a)

Without loss of generality, following the completion of any journey comprising *n* steps, we can reverse the order of the step indices such that step *m* is now labelled step *n-m+1*. Thus step *n* is relabelled step *1*, and step *1* relabelled step *n*. Equation S1.7a can be rewritten as

$\left[\begin{matrix} U'_{n-m+1} \\ V'_{m-m+1} \end{matrix}\right]=-\mathbf{R}\left(-\sum_{i=1}^{n-m+1} \delta_{i}\right)\left[\begin{matrix} \Lambda_{n-m+1} \\ 0 \end{matrix}\right]+\mathbf{R}\left(-\sum_{i=1}^{n-m+2} \delta_{i}\right)\left[\begin{matrix} \varepsilon_{n-m+1}^{U'} \\ \varepsilon_{n-m+1}^{V'} \end{matrix}\right]+\left[\begin{matrix} \varepsilon_{1}^{U'} \\ \varepsilon_{1}^{V'} \end{matrix}\right]$ (S1.7b)

From S1.7b, and letting $j=n-m+1$, S1.7 can be rewritten as

$\left[\begin{matrix} U'_{n} \\ V'_{n} \end{matrix}\right]_{Total}\Leftrightarrow\left[\begin{matrix} U'_{1} \\ V'_{1} \end{matrix}\right]_{Total}=-\sum_{j=1}^{n} \mathbf{R}\left(-\sum_{i=1}^{j} \delta_{i}\right)\left[\begin{matrix} \Lambda_{j} \\ 0 \end{matrix}\right]+\sum_{j=2}^{n} \mathbf{R}\left(-\sum_{i=1}^{j+1} \delta_{i}\right)\left[\begin{matrix} \varepsilon_{j}^{U'} \\ \varepsilon_{j}^{V'} \end{matrix}\right]+\left[\begin{matrix} \varepsilon_{1}^{U'} \\ \varepsilon_{1}^{V'} \end{matrix}\right]$ (S1.7c)

where is used to denote quantitative equivalence following index relabeling.

Using an EDVR (e.g. Fig. S1C),

$\begin{matrix} \left[\begin{matrix} U'_{n} \\ V'_{n} \end{matrix}\right]_{Total}=\mathbf{R}\left(\varepsilon_{n}\right)\mathbf{R}\left(-\delta_{n}\right)\left(\left[\begin{matrix} U'_{n-1} \\ V'_{n-1} \end{matrix}\right]_{Total}+\left[\begin{matrix} -\Lambda_{n} \\ 0 \end{matrix}\right]\right) \\ =\mathbf{R}\left(\varepsilon_{n}-\delta_{n}\right)\left(\mathbf{R}\left(\varepsilon_{n-1}-\delta_{n-1}\right)\left(\left[\begin{matrix} U'_{n-2} \\ V'_{n-2} \end{matrix}\right]_{Total}+\left[\begin{matrix} -\Lambda_{n-1} \\ 0 \end{matrix}\right]\right)+\left[\begin{matrix} -\Lambda_{n} \\ 0 \end{matrix}\right]\right) \\ =\cdots=-\sum_{j=1}^{n} \mathbf{R}\left(\sum_{i=j}^{n} \left(\varepsilon_{i}-\delta_{i}\right)\right)\left[\begin{matrix} \Lambda_{j} \\ 0 \end{matrix}\right] \end{matrix}$ (S1.8)

Again, we can relabel the step indices as per the ESVR case to obtain a quantitatively equivalent expression which follows the form of an IDW (S1.4).

$\left[\begin{matrix} U'_{n} \\ V'_{n} \end{matrix}\right]_{Total}\Leftrightarrow\left[\begin{matrix} U'_{1} \\ V'_{1} \end{matrix}\right]_{Total}=-\sum_{j=1}^{n} \mathbf{R}\left(\sum_{i=1}^{j} \left(\varepsilon_{i}-\delta_{i}\right)\right)\left[\begin{matrix} \Lambda_{j} \\ 0 \end{matrix}\right]$ (S1.8a)

Clearly, only PI using an ASVR results in a recorded path in representational space which resembles an ADW (the rest behave as IDWs). Since the expected trajectory of an ADW is always proportional to the ideal path, and is generally associated with relatively small positional uncertainty, ASVRs are plausible candidates for biological PI. In contrast, the expected trajectory of an IDW has a limited length irrespective of the number of steps taken, is nonlinearly related to the ideal path, and is generally associated with large positional uncertainty, ADVRs, ESVRs, and EDVRs are much less plausible candidates for biological PI.

A long tortuous path can be considered as a series of shorter approximately straight segments. This guarantees the results reported here are applicable to any navigation journey, in either real or representational space. Note also that the update error for radial measures e.g., $\varepsilon_{m}^{r}$ and $\varepsilon_{m}^{r'}$*’* have been omitted for clarity. These linear random error terms can be considered as a scaling of current and previous step lengths in representational space. For an ideal step length Λ_0_ in representational space,

$\Lambda_{m}=\Lambda_{0}\prod_{j=1}^{m} \frac{r_{j}+\varepsilon_{j}^{r}}{r_{j}}$ (S1.9)

but for directed journeys (e.g. modelled as IDWs or ADWs), *r* increases without bound thus

$\lim_{j\to\infty} \frac{r_{j}+\varepsilon_{j}^{r}}{r_{j}}=1$ (S1.10)

Furthermore, since

$\Lambda_{m}=\left(1+\frac{\varepsilon_{m}^{r}}{r_{m}}\right)\Lambda_{m-1}=\left(1+\frac{\varepsilon_{m}^{r}}{r_{m}}\right)\prod_{j=1}^{m-1} \left(1+\frac{\varepsilon_{j}^{r}}{r_{j}}\right)$ (S1.11)

and we assume *ε_j_* and *ε_j+1_* are random errors which are statistically independent,

$\begin{matrix} \left\langle\Lambda_{m}\right\rangle=\left\langle\left(1+\frac{\varepsilon_{m}^{r}}{r_{m}}\right)\Lambda_{m-1}\right\rangle=\left\langle\Lambda_{m-1}\right\rangle+\left\langle\frac{\varepsilon_{m}^{r}}{r_{m}}\Lambda_{m-1}\right\rangle=\left\langle\Lambda_{m-1}\right\rangle+\left\langle\frac{\varepsilon_{m}^{r}}{r_{m}}\right\rangle\left\langle\Lambda_{m-1}\right\rangle\\ =\left\langle\Lambda_{m-1}\right\rangle=\cdots=\left\langle\Lambda_{1}\right\rangle=\Lambda_{0} \end{matrix}$ (S1.12)

Therefore, there is no distortion of the ideal step size in representational space, merely an increase in variance. Since we already know from directed walk theory that random errors in step size are of little consequence when cumulative angular errors occur, we are justified in neglecting this updating error in considering (A/E)DVRs such as polar representations.
