# Supplementary material for: Finding the Way with a Noisy Brain
Source: PLoS Comput Biol. 2010 Nov 11;6(11):e1000992. doi: 10.1371/journal.pcbi.1000992 (PMC2978673; doi:10.1371/journal.pcbi.1000992)
Supplement: Text S2 — A special case: using compass readings to estimate rotations. (0.06 MB DOC) [file pcbi.1000992.s005.doc]

**S2 A Special Case: Using Compass Readings to Estimate Rotations**

Previously, we assumed an independent error value *δ* was added to each measurement of angular displacement or rotation . If we consider the actual heading following *n* steps obtained via an accumulation of such noisy turns, the result is the correct heading plus *n* independent errors i.e.,

(S2.1)

However, there is a different consequence if the following three criteria are met. Firstly, assume that the *n-1*’th noisy compass reading is used as the current heading for calculating the *n-1*’th rotation and stored temporarily to become the reference direction for calculating the *n*’th rotation, and so on for every step. Secondly, the process whereby the current noisy direction becomes the next noisy reference direction is itself noise-free. Thirdly, the process whereby the calculated rotation is transformed for HV updating is also entirely free from error. In essence, the only angular noise allowed is during the compass reading, and the noisy measurements are preserved exactly thereafter, with no further source of random error. Algebraically, this scheme results in the following heading after *n* steps:

(S2.2)

Interestingly, the magnitude of the heading error is independent of *n* and therefore won’t accumulate. We consider this to be more of a mathematical curiosity than biological possibility, but consider it here for completeness.

For DVRs, an egocentric representation is less stable than an allocentric one since the update error, ε^θ^, alone is sufficient to cause path degenerescence and map a straight trajectory in real space to an IDW in representational space during PI. However, for ESVR, in the unlikely situation that all three conditions listed earlier are met simultaneously, then it is theoretically possible to avoid IDW-like behaviour of the representational path during PI. Nonetheless, there is still one further issue related to egocentric representations which makes it inferior to allocentric representations in the presence of noise. The required change in egocentric static vectorial coordinates scale linearly with the radial distance from the starting point of the journey [7]. Hence any heading error will have the same effect. For example, suppose we rewrite the noisy EC update equations from Table S2,

In simulated journeys using these equations, the representational path during PI appears to stay closer to the true path than all but members of the ASVR class (data not shown). What is the reason for this?

For simplicity, consider an outbound path which is perfectly straight. Since the variance of the independent update errors *ε* sum linearly, they contribute little to the overall positional uncertainty compared to the rotational error *δ*. Following *n* steps, and for small *δ*, the variance of *V_n_* is

$V\left(V_{n}\right)\tilde{\propto}V\left(r'\theta'\right)=r'^{2}V\left(\delta\right)\propto n^{2}\sigma_{\delta}^{2}$ (S2.3)

A straight trajectory in real space recorded by this hypothetical EC PI system maps approximately to

$\left[\begin{matrix} U'_{n} \\ V'_{n} \end{matrix}\right]\approx r'_{n}\left[\begin{matrix} \cos\left(-\delta_{n}\right) \\ \sin\left(-\delta_{n}\right) \end{matrix}\right]$ (S2.4)

in representational space (apostrophe denotes egocentric coordinates following the convention of [7]). Now, the positional variance of an ADW increases linearly with the step number *n*, whereas the positional variance of (*U’_n_*, *V’_n_*) increases as *n^2^*. Therefore, even assuming the input noise is relatively small, this hypothetical EC PI system seems to be inferior to an ASVR. We expect this property to apply to all ESVRs.
