# Supplementary material for: Finding the Way with a Noisy Brain
Source: PLoS Comput Biol. 2010 Nov 11;6(11):e1000992. doi: 10.1371/journal.pcbi.1000992 (PMC2978673; doi:10.1371/journal.pcbi.1000992)
Supplement: Text S3 — Allocentric static vectorial representations. (0.14 MB DOC) [file pcbi.1000992.s006.doc]

**S3 Allocentric Static Vectorial Representations**

An exact, and an approximate (but asymptotically exact) update method using a vectorial basis consisting of (non-parallel) unit vectors radiating from the origin has been reported [7]. Both are examples of an ASVR. They are briefly reviewed here and then noise is added to demonstrate an analytical equivalence between members of this class. Finally, we consider how even an ASVR may succumb to (systematic) error.

In the simplest example of the first case, **v** may be represented by a linear combination of the two adjacent unit vectors **u_j_** and **u_j+1_** so that $\mathbf{v}=a_{j}\mathbf{u}_{\mathbf{j}}\mathbf{+}a_{j+1}\mathbf{u}_{\mathbf{j+1}}$. Upon careful consideration of the vectorial geometries, it can be shown that and , where *θ* is the allocentric angle of the static vector in question,  is the direction of **v**, and Λ is the length of **v**. Fig. 5A and 5B shows two different examples of static vectorial representations with variable amplitudes, and the projections of the exact scalar amplitudes *a_j_* and *a_j+1_* of adjacent static vectors **u_j_** and **u_j+1_** to exactly equal **v**.

Now consider the addition of random noise to the PI system.

(S3.1)

where $\varepsilon_{X}=\varepsilon_{j}\cos\theta_{j}+\varepsilon_{j+1}\cos\theta_{j+1}$ and $\varepsilon_{Y}=\varepsilon_{j}\sin\theta_{j}+\varepsilon_{j+1}\sin\theta_{j+1}$. This is similar to the standard form of a step in representational space using a simple ASVR (see Table S1). The major difference is that

$Cov\left(\varepsilon_{X},\varepsilon_{Y}\right)=\left\langle\varepsilon_{X}\varepsilon_{Y}\right\rangle-\left\langle\varepsilon_{X}\right\rangle\left\langle\varepsilon_{Y}\right\rangle=\sigma_{\varepsilon}^{2}\left(\sin\left(2\theta_{j}\right)+\sin\left(2\theta_{j+1}\right)\right)$ (S3.2)

Hence $Cov\left(\varepsilon_{X},\varepsilon_{Y}\right)\geq0$ with equality iff $\theta_{j+1}=\theta_{j}\pm\pi/2$, which is then a Cartesian representation. In general, there is a positive covariance term. However, this is of little consequence since the error associated with the estimate of step size *L* and the angular error Δ may be statistically dependent in any conceivable way and the resulting path is still an ADW so long as Δ does not accumulate between steps [21]. In an analogous fashion, *ε_X_* and *ε_Y_* may be considered part of the arbitrarily complex variability within each unit of locomotion – only here it is in representational space. Taken together, these results support the notion that all static vectorial representations should be considered as a single class. Furthermore, for any given PI behaviour, if one static vectorial representation is an adequate explanation, then others of this class are also.

Suppose there are a large number of static vectors, and each one suffered from an independent update error *ε* during every step, irrespective of the heading . Then

$\begin{matrix} Cov\left(\varepsilon_{X},\varepsilon_{Y}\right)=\left\langle\varepsilon_{X}\varepsilon_{Y}\right\rangle=\left\langle\left(\sum_{j=1}^{\nu} \varepsilon_{j}\cos\theta_{j}\right)\left(\sum_{j=1}^{\nu} \varepsilon_{j}\sin\theta_{j}\right)\right\rangle\\ =\cdots=\sum_{j=1}^{\nu} \left\langle\varepsilon_{j}^{2}\right\rangle\sin\left(2\theta_{j}\right)\tilde{\propto}\sigma_{\varepsilon}^{2}\int_{0}^{2\pi} \sin\left(2\theta\right)d\theta=0 \end{matrix}$ (S3.3)

This may be a more appropriate description of the update errors of the ASVR architecture described below. It shows that when linear update errors, *ε*, occur in all allocentric directions, the linear covariability of *ε_X_* and *ε_Y_* vanishes due to symmetry. Again, the result is that update errors cause positional displacement but not heading displacement. Therefore, a straight trajectory in real space will map to an ADW in representational space, like other implementations of ASVRs.

An alternative updating mechanism involves a transfer function *f* which, like a tuning curve, has a variable response which depends on the current heading  (see blue arrows in Fig. 5B). The HV representation requires a ring of *ν* neural units, each with transfer function *f* which is offset by $2\pi/\nu$ radians from the adjacent one. Thus . Then the HV update equation for axis *j* is . This approximate update method is asymptotically exact as the number of static vectors increase [7].

Consider a continuous approximation of the ring when *ν* is very large so that $2\pi/\nu\to0$, then we can examine the general transfer function in the following way. In the *j*-centric coordinates of axis *j*, we have

$x_{j}'=\int_{-\pi}^{\pi} f\left(\theta\right)\cos\left(\theta\right)d\theta$ (S3.4)

$y_{j}'=\int_{-\pi}^{\pi} f\left(\theta\right)\sin\left(\theta\right)d\theta$ (S3.5)

If *f* is unbiased, then by definition, $y_{j}'=0$ and it is obvious that the HV representation is perfect. However, it can be shown that in the absence of noise, as long as $x_{j}'>0$ the HV representation will still allow successful homing, even if there is a systematic angular error in the sensorimotor loop. Thus, a true heading of  might actually correspond to a recorded direction of *+ζ*. Suppose the animal maintained that trajectory for some time, its PI system registering that it has travelled some distance from home in allocentric direction *+ζ*. At the beginning of homing, according to its HV, it rotates to *+ζ+π* which is *ζ* radians too much. As it moves in this direction, its PI registers locomotion in allocentric direction *+2ζ+π*. To compensate, the new HV rotates in the opposite direction to *ζ* and so on for all steps until home is reached.

The proof of ‘guaranteed’ successful homing is intuitive when the problem is framed in polar coordinates. Firstly, there is a systematic angular error $\zeta=\tan\left(y_{j}'/x_{j}'\right)$ during the HV updating process. Since this error is a characteristic of *f* and independent of the rate of movement *s* or heading , its effect is to rotate each step, and hence the entire HV representation, by exactly $\zeta$ during the outbound journey. During homing, assume for simplicity that steering is instantaneous, then the navigating agent always points in the direction its HV is pointing. At the beginning of the homeward journey, its heading will be $\zeta$ away from the true home direction, at distance *r_0_*. However, every step during homing results in a displacement which is also rotated by $\zeta$ since the same *f* and HV updating process are in use. Consequently, no matter how many steps are taken, the HV must still be $\zeta$ away from the true home direction. It is clear that if $\zeta=\pi/2$, the path will approximate a circle with radius *r_0_*. If $\zeta>\pi/2$ the path approximates an outward logarithmic spiral and the navigating agent will never reach home. If $\zeta<\pi/2$ the path approximates an inward logarithmic spiral converging on home. In continuous time, the total homeward path length (excluding noise and searching) is $r_{0}\sqrt{1+\tan^{2}\left(\zeta\right)}$. There is a surprising tolerance to systematic angular error in the transfer function *f* e.g. $\zeta=\pi/3$ only results in doubling of the total homeward path length.

The corollary of the above argument is that as long as any systematic noise in the transfer function *f* results in less than a 90° bias, then the mean homeward trajectory will be an inward logarithmic spiral during PI using an ASVR. However, if the systematic bias exceeds 90°, then even an ASVR cannot sustain PI.

It should be noted that the logarithmic spirals described here are distinct from the general expected path of an IDW [21]. In the former case, the curvature of the trajectory is due to a net discrepancy between true heading and represented heading, resulting in a bias from the true homeward direction. In the latter case, the curvature results from a net angular bias in locomotion, accumulating with each step. Furthermore, the expected displacement per step diminishes exponentially in an IDW, whereas the expected displacement during PI homing using an ASVR is actually constant throughout the journey. The last property actually makes the approximation of the logarithmic spiral superior for the general IDW than PI homing via a biased ASVR.

Note: For this work, it is not important whether there is a direct correspondence of each algebraic quantity with a single definable characteristic/property of a neuron. However, for ease of conceptualization, one might consider each unit vector **u**_j_ to have an associated neuron, and the amplitude *a_j_* may be represented by the state of that neural unit, e.g. its action potential firing rate, or its synaptic weight on another neuron.
